# Trends over time in the deficit of (instrumental) activities of daily living in the Austrian population aged 65 years and older: Results from the Austrian Health Interview Survey series

**DOI:** 10.1007/s00508-024-02388-4

**Published:** 2024-06-18

**Authors:** Selam Woldemariam, Viktoria K. Stein, Sandra Haider, Thomas E. Dorner

**Affiliations:** 1 Karl Landsteiner Institute for Health Promotion Research, Kirchstetten, Austria; 2 Center for Public Health, Department for Social and Preventive Medicine, Medical University of Vienna, Vienna, Austria; 3 Academy for Ageing Research, “Haus der Barmherzigkeit”, Vienna, Austria

**Keywords:** Disabilities prevalence, Mobility, Self-care, Long-term care, Health promotion

## Abstract

**Background:**

Difficulties in activities of daily living (ADL) and instrumental activities of daily living (IADL) in older adults are associated with diminished quality of life and increased demand for long-term care. The present study examined the prevalence of disability among individuals aged 65 years and older in Austria, using data from the Austrian Health Interview Surveys (ATHIS).

**Methods:**

The ATHIS 2014 and 2019 surveys were used (*N* = 5853) for the analysis. Binary logistic regression was performed to measure the association between disability in at least one ADL or IADL limitation and independent variables adjusted for sociodemographic, health-related behavior and survey year.

**Results:**

The prevalence of ADL or IADL limitations increased in both sexes during the 5‑year follow-up period. For ADL limitations, the prevalence rose from 12.8% to 17.9% in men (*p* < 0.001) and from 19.2% to 25.7% in women (*p* < 0.001). The IADL limitations increased from 18.9% to 35.1% in men (*p* < 0.001) and from 38.2% to 50.8% in women (*p* < 0.001). Women reported significantly higher odds for ADL (odds ratio [OR]: 1.08, 95% confidence interval [CI]: 0.93–1.26) and IADL limitations (OR: 1.74, 95% CI: 1.53–1.98). In both sexes, participants aged 80 years and older reported higher odds for ADL (OR: 4.37, 95% CI:3.77–5.07) and IADL limitations (OR: 4.43, 95% CI: 3.86–5.09) compared to the younger group. Participants with at least one chronic disease reported higher odds for ADL (OR: 4.00, 95% CI: 3.41–4.70) and IADL limitations (OR: 4.37, 95% CI: 3.85–4.96). Primary education, single status, being born in non-EU/EFTA countries, and residing in Vienna were associated with higher odds of ADL and IADL limitations.

**Conclusion:**

Gender, age, education, country of birth, residence, partnership status, number of chronic diseases, noncompliance with physical activity, and nutrition recommendations had a strong association with increased vulnerability to disability. Public health policy must address these factors for disability prevention strategies.

## Introduction

As people live longer, crucial questions arise about how the prevalence of disabilities is developing over time in older populations. Can older adults maintain functional independence despite age-related conditions like frailty, which increases their vulnerability to adverse health outcomes? Frailty is associated with an increased risk of falls, hospitalisation, disability, and mortality, thus influencing the transition from healthy ageing to disability [1–3]. Today, the question of older adult functional health is particularly relevant in Europe due to the ongoing demographic shifts and their potential impact on future healthcare demands, both formal and informal, and the provision of long-term care for the growing ageing population.

Several studies have examined disability prevalence and quality of life to gain insights into the health status of older adults. Research shows that limitations in activities of daily living (ADL) and instrumental activities of daily living (IADL) are associated with low quality of life [4, 5], poor health [6], and increased risk of mortality [7], indicating additional years of life spent in poor health. Furthermore, the increasing trend in disability prevalence in European countries among individuals aged 65 years and older highlights the potential need for increased support and long-term care for older populations [8–12]. Notably, variations in disability prevalence have been observed according to gender, with women being more vulnerable to disabilities compared to men [11, 12]. However, a study based on community-dwelling older adults in the UK observed a decline in the prevalence of functional impairments among men but not women. These conflicting results are likely attributed to variations in disability measurement used in the study [13].

Furthermore, conflicting findings in disability prevalence have been observed between population sub-groups. More recently, studies in the UK have examined the burden of disability according to socioeconomic characteristics to identify disparities in disability among older adults [14, 15]. A nationally representative survey from Finland [16] found a robust association between lower education levels and increased disability prevalence, even after controlling for urbanisation, chronic diseases, depressive symptoms, and the survey period. Moreover, findings from cohort-specific effects on disability trends indicate that recently born cohorts have flat trajectories in disability prevalence compared to older cohorts [12, 13]. Factors contributing to the variation in disability prevalence, even when controlling for age and other characteristics, may be due to advances in medicine, increased access to education, and public health programs. Similarly, a study from Norway [17] found no overall improvement in physical function but reported an improvement in cognitive function in later cohorts, assessed through fluency, and immediate and delayed recall measures. The authors argue that this cognitive improvement is linked to increased education levels, which may influence lifestyle behavior, the burden of chronic diseases, and overall living conditions, subsequently delaying the onset of disability.

The association between chronic conditions and ADL and IADL limitations suggests that older adults with chronic diseases experience a steeper increase in functional limitations [18–20]. A retrospective cohort study conducted in Taiwan [15] further examined the burden of disease-related disability and found that ADL/IADL trajectories were highest in study participants diagnosed with diabetes mellitus, cancer, and hypertension. These findings highlight that although mortality may be averted, the presence of chronic diseases and associated risk factors may lead to an earlier onset of disability. Therefore, maintaining good health through lifestyle behaviours is crucial for individuals and society.

Evidence on the prevalence of ADL/IADL limitations and trends over time is scarce and ambiguous in many European countries, including Austria. Therefore, the present study aims to examine the prevalence of disability over 5 years in Austrian adults aged 65 years and older using data from the Austrian Health Interview Surveys (ATHIS) from 2014 and 2019.

## Method

The present study used data from two waves conducted in 2014 and 2019 from the Austrian Health Interview Survey (ATHIS) series [21, 22]. As part of the European Health Interview Survey (EHIS) framework, the ATHIS is a nationwide survey designed to collect information on the population’s health status, health determinants, and socioeconomic background of private households in Austria [23]. The target population was individuals aged 15 years and older, registered in Austria’s national central population register. The population was stratified into 32 geographical regions and for the three regions covering Vienna, and the target was 560 and 575 participants for ATHIS 2014 and 2019, respectively [21, 22]. In sparsely populated regions, a minimum of 300 participants were included in both surveys [21, 22]. Both cross-sectional waves were conducted independently. ATHIS 2014 was carried out via computer-assisted telephone interviewing (CATI) from October 2013 to June 2015, while ATHIS 2019 was carried out using a combination of computer-assisted personal interviewing (CAPI) and a web-based questionnaire from October 2018 to September 2019. For the physical activity (PA) questionnaire, participants who did not respond to the self-administered survey received a paper questionnaire via post to participate in CATI or CAPI [21, 22]. Net sample sizes for the two waves were 15,771 and 15,461 persons, respectively, with response rates of 40.7% and 50.5% in the respective waves.

To measure disability, self-reported limitations in ADL from Katz et al. [24] and IADL from Lawton and Brody [25] were used. The ADL index consisted of five items that assessed whether participants had difficulties with eating or drinking, getting into/out of bed, dressing, using the toilet, and bathing or taking a shower. The IADL index included seven items that assessed whether participants had difficulties with preparing meals, using the telephone, shopping, managing medication, undertaking light housework, undertaking heavy housework, and managing money [21, 22]. The corresponding question was, “Do you usually have difficulty doing any of the following activities by yourself without help?”. The response categories for both ADL and IADL indices were “No difficulty”, “Some difficulty”, “A lot of difficulty” and “Cannot do at all/unable to do by myself”, with the latter three combined into a single category “yes” for our analysis.

The following sociodemographic data were collected: sex (male, female), age (65–79, 80 years and over), education (primary, secondary, and tertiary) were grouped according to the International Standard Classification of Education (ISCED) [26], country of birth (Austria, EU or EFTA states, and non-EU/EFTA states), region of residence (Vienna, other federal states), partnership status (married or in a relationship—yes or no). Chronic disease presence was obtained by the question, “Do you have a long-term illness or chronic health problem?” with the response option yes or no, to gather participants’ health conditions in the last 6 months [21, 22]. Additionally, body mass index (BMI) was calculated from self-reported body weight and height and classified as follows: underweight (BMI < 18.5 kg/m^2^), normal (BMI 18.5–24.9 kg/m^2^), overweight (BMI 25–29 kg/m^2^) or obese (BMI ≥ 30 kg/m^2^) [27].

Compliance with PA recommendations was measured according to the European Health Interview Survey—Physical Activity Questionnaire (EHIS-PAQ) [28] to calculate the weekly minutes participants spend cycling, playing sports, or participating in fitness or leisure physical activities, aggregating moderate and vigorous intensity activities. Both the international and Austrian PA guidelines advise at least 150–300 min per week of moderate intensity aerobic PA, 75–150 min per week of vigorous intensity PA, or an equivalent combination of both, in addition to at least two sessions per week of muscle-strengthening PA [29, 30]. As such, the responses from the EHIS-PAQ were dichotomized to indicate compliance with both endurance and strength-related PA criteria or noncompliance. Compliance with nutrition recommendations was measured with the question, “How many portions of fruits and vegetables, do you eat per day?” [21, 22]. The daily nutrition recommendations are at least five portions of fruits or vegetables, with an emphasis on vegetable consumption [31]. We dichotomized participants’ responses to reflect compliance or noncompliance according to the European dietary guidelines.

All analyses were restricted to participants aged 65 years and over. The study sample was weighted using the geographical region, age in 5‑year groups, sex, family status, migration background, and educational level, as the weighting factors. Bivariate analyses were computed with cross-tabulations, to assess the proportion of individuals within sociodemographic characteristics across survey waves. Group differences were evaluated using Pearson’s χ^2^-tests. Finally, binary logistic regression analyses were performed with disability in at least one ADL or IADL limitations as the dependent variable, and survey year, sociodemographic and health-related parameters as the independent variables. The estimates of the logistic regression models were mutually adjusted for sociodemographic and health-related variables and are presented as an odds ratios (OR) and 95% confidence intervals (CI). All statistical analyses were conducted using IBM SPSS Statistics Version 29 (IBM Corporation, Armonk, NY, USA).

The secondary analysis of the ATHIS database was approved by the Ethics Committee of the Medical University Vienna (EK # 2211/2015 for ATHIS 2014 and EK #1263/2021 for ATHIS 2019).

## Results

A total of 5853 participants were included in the analysis, and Table 1 illustrates the characteristics of the samples from both surveys. Among the study participants, there was a slightly higher percentage of females in both survey waves, with most of them in the 65–79 years age group. Many of the participants in both survey waves had completed secondary education, were born in Austria, resided in federal states outside Vienna, and were living in a partnership. More than half of the study participants had reported at least one chronic condition and almost two thirds of the participants were either overweight or obese. A significant proportion of participants did not comply with the recommended guidelines for both PA and nutrition. Between 2014 and 2019, there was a significant increase in the proportion of participants who had completed secondary education, were born outside of the non-EU/non-EFTA region, reported more than one chronic disease, and did not comply with PA or nutrition recommendations.

**Table 1:** Participant characteristics from the ATHIS 2014 and 2019 surveys

	2014	2019	*P*-value^a^
	*N* = 2934	*N* = 2919	
*Sex*			
Male	43.5	44.3	0.505
Female	55.5	55.7	–
*Age (years)*			
65–79	74.8	74.4	0.713
≥ 80	25.2	25.6	–
*Education level*			
Primary	35.4	31.9	0.010
Secondary	56.1	59.3	–
Tertiary	8.5	8.8	–
*Country of birth*			
Austria	85.6	87.7	< 0.001
EU/EFTA	10.8	7.7	–
Non-EU/non-EFTA	3.6	4.7	–
*Region of residence*			
Vienna	18.7	18.7	0.988
Other federal states	81.3	81.3	–
*Partnership status*			
In a relationship/married	60.7	60.5	0.861
Not in a relationship/married	39.3	39.5	–
*Self-reported chronic disease*			
≥ 1 chronic disease	54.1	59.9	< 0.001
No chronic disease	45.9	40.1	–
*BMI*			
Normal weight	37.6	35.9	0.048
Overweight	41.0	43.2	–
Obese	19.6	19.7	–
Underweight	1.8	1.1	–
*PA recommendations*^b^			
Compliance	22.5	12.1	< 0.001
Noncompliance	77.5	87.9	–
*Nutrition recommendations*^c^			
Compliance	6.1	3.6	< 0.001
Noncompliance	93.9	96.4	–

Values are given as percentage (%)

^a^*P*-values are result of the χ^2^-tests between 2014 and 2019.

^b^PA recommendations: 150–300 min/week PA with moderate intensity, or 75–150 min/week PA with vigorous intensity, or an equivalent combination of the two, PLUS at least 2/week muscle strengthening PA.

^c^Nutrition recommendations: At least 5 servings of fruit or vegetables per day

The results presented in Table 2 show the prevalence of ADL and IADL limitations among study participants in the 2014 and 2019 surveys. In the 2014 survey, 68% of participants aged 65 years and over reported no ADL limitations, while 42.9% reported no IADL limitations. Conversely, in the 2019 survey, these percentages decreased, with only 42% reporting no ADL limitations and 14.1% reporting no IADL limitations. Overall, the prevalence of ADL or IADL limitations increased in both sexes from the 2014 to 2019 survey, and this significant increase in limitations could be observed in every ADL and IADL domain. Specific to ADL tasks, the most frequent limitations in both sexes were observed in the activities of transferring from bed to chair; in 2019, almost 13.3% of men and 20.4% of women reported restriction in this task. For IADL tasks, activities in heavy housework were the most reported limitation. Among the 90.4% of the participants who experienced limitations in at least 1 activity of ADL tasks, 41.2% also reported difficulties in IADL limitations. In general, across both survey periods, participants of both sexes reported an 85.9% higher rate of IADL limitations compared to 43.6% for ADL limitations in the 2019 survey waves. Overall, in both survey years, women consistently reported a higher proportion of limitations in both ADL and IADL tasks.

**Table 2:** Proportion of participants with ADL and IADL limitations by gender

		Men			Women	
	2014	2019		2014	2019	
	*N* = 1423	*N* = 1508	*P-*value^a^	*N* = 1511	*N* = 1411	*P-*value^a^
*Limitation with at least one ADL*	12.8	17.9	< 0.001	19.2	25.7	< 0.001
Eating or drinking	1.0	3.8	< 0.001	1.7	8.7	< 0.001
Transfer (from bed to chair)	8.8	13.3	< 0.001	12.6	20.4	< 0.001
Dressing	5.8	12.6	< 0.001	8.9	18.7	< 0.001
Toileting	2.2	6.7	< 0.001	4.0	11.9	< 0.001
Bathing or showering	5.5	11.3	< 0.001	11.1	19.9	< 0.001
*Limitation with at least one IADL*	18.9	35.1	< 0.001	38.2	50.8	< 0.001
Preparing meals	2.4	10.3	< 0.001	9.7	18.6	< 0.001
Using the telephone	2.2	7.9	< 0.001	3.4	11.2	< 0.001
Shopping	6.7	13.1	< 0.001	14.8	26.2	< 0.001
Managing medication	1.7	9.2	< 0.001	3.1	15.6	< 0.001
Doing light housework	4.7	13.5	< 0.001	9.0	22.8	< 0.001
Doing heavy housework	14.3	30.7	< 0.001	33.0	48.0	< 0.001
Managing money	3.5	11.7	< 0.001	8.3	20.7	< 0.001

Values are given in percentages (%)

^a^*P*-values are result of the χ^2^-tests between 2014 and 2019.

Table 3 shows the association between the sociodemographic and health-related factors with ADL and IADL limitations. In the adjusted model, there was a significantly higher chance of experiencing ADL and IADL problems in the ATHIS 2019 cohort compared with the ATHIS 2014 cohort, with a 32% higher odds in ADL and 94% higher odds in IADL limitations in the ATHIS 2019 cohort. There were significantly higher odds for ADL and IADL limitations among females and participants in older age groups (80+ years). Moreover, those with primary education, not in a relationship, born outside the EU/EFTA regions, and residing in Vienna had significantly higher odds of ADL and IADL limitations. Furthermore, having at least one chronic disease was also associated with 4.00- and 4.37-fold higher odds in both cohorts, respectively, in comparison to those without chronic conditions. Furthermore, participants who were noncompliant with PA and daily nutrition recommendations had significantly higher odds of ADL and IADL limitation in both cohorts.

**Table 3:** Association between sociodemographic and health-related factors with ADL and IADL limitations in the pooled datasets of ATHIS 2014 and 2019

	ADL limitations	IADL limitations
	OR (95% CI)	OR (95% CI)
*Survey year*		
2014	1	1
2019	1.32 (1.15–1.52)	1.94 (1.72–2.19)
*Sex*		
Male	1	1
Female	1.08 (0.93–1.26)	1.74 (1.53–1.98)
*Age (years)*		
65–79	1	1
≥ 80	4.37 (3.77–5.07)	4.43 (3.86–5.09)
*Education level*		
Primary	1.86 (1.38–2.51)	2.47 (1.93–3.17)
Secondary	1.12 (0.83–1.49)	1.36 (1.07–1.72)
Tertiary	1	1
*Country of birth*		
Austria	1	1
EU/EFTA	0.83 (0.65–1.06)	0.94 (0.76–1.15)
Non-EU/Non-EFTA	1.82 (1.33–2.48)	1.31 (0.98–1.75)
*Region of residence*		
Vienna	1.08 (0.90–1.29)	1.27 (1.09–1.48)
Other federal states	1	1
*Partnership status*		
In a relationship/married	1	1
Not in a relationship/married	1.61 (1.38–1.85)	1.32 (1.16–1.49)
*Self-reported chronic disease*		
No chronic disease	1	1
≥ 1 chronic disease	4.00 (3.41–4.70)	4.37 (3.85–4.96)
*BMI*		
Normal weight	1	1
Overweight	1.20 (1.02–1.41)	1.15 (1.00–1.32)
Obese	2.02 (1.67–2.44)	1.75 (1.48–2.06)
Underweight	1.11 (0.66–1.85)	2.22 (1.35–3.66)
*PA recommendations*		
Compliance	1	1
Noncompliance	1.96 (1.53–2.50)	2.19 (1.82–2.93)
*Nutrition recommendations*		
Compliance (5 portions/day)	1	1
Noncompliance	2.55 (1.65–3.93)	1.43 (1.07–1.91)
R^2^	0.282	0.355

Result of a binary regression analysis, all variables are mutually adjusted for each other. Results are shown in OR (95% CI)

## Discussion

The nationally representative data in our study revealed a general increased trend in ADL and IADL limitations among adults aged 65 years and older. Over the 5‑year study period, a significant 32% increase in the risk of ADL disabilities and a substantial 94% increase in IADL disabilities were observed. The study findings also indicate that the increased risk of ADL and IADL limitations varied according to several factors, including gender, age, education, country of birth, residence, partnership status, body weight, number of chronic diseases and lifestyle behaviors.

In the 80-year-old and older age group our findings demonstrated a significant increase in ADL and IADL limitations, aligning with similar patterns observed in previous studies in various European countries [8–10, 32, 33] and the US [34]. In general, women were most affected and reported a higher prevalence of ADL and IADL limitations compared to men, consistent with previous studies [11, 12, 34]. A plausible explanation for these sex differences may be linked with lower muscle mass associated with sex hormones in women, which are significantly more pronounced age-related declines in muscle mass in women compared to men [2]. The higher disability prevalence in women may also be linked to sex differences in physical activity. A study of 1845 Australian adults aged 60 and above found that women tend to be less active in aerobic exercise compared to men [35]. These physiological differences likely contribute to the observed variations, emphasizing the pivotal role of sex/gender in influencing disability outcome and the importance in developing targeted gender-specific interventions in long-term care.

Multimorbidity, indicated by an increase in the number of chronic diseases, was associated with an increased risk of disability in both ADL and IADL tasks, confirming patterns found in previous studies [18, 19, 36]. While our analysis did not explore specific chronic diseases and their association with disability, a Canadian study [37] identified chronic conditions, such as arthritis and heart problems, as having the most significant impact on ADL and IADL functional disabilities in similar age cohort groups. Nevertheless, in our study, a strong association was observed between body weight and the risk for disability. Our study results showed that a higher BMI increases the risk of ADL and IADL limitations. A similar finding was observed in the US [38], where clinically obese participants (BMI ≥ 35 kg/m^2^) were associated with an increased prevalence of ADL limitations by 18% for men and 22% for women. Interestingly, our study also showed a twofold risk for IADL deficits in underweight participants compared to those with normal weight. Previous research also indicates that underweight older adults can face an increased risk of disabilities due to inadequate nutrition [39, 40]. This association may be attributed to the fact that underweight is often associated with undernutrition and energy-protein malnutrition (PEM) [39]. Consequently, PEM and weight loss are risk factors in older adults for osteoporotic fractures and sarcopenia, which are characterized by loss of muscle strength and function, thereby negatively impacting the maintenance of cognitive and physical function [39–42]. This physical and functional impairment among malnourished participants indicates that due to their body weight, participants are experiencing activity restriction in everyday life, thus highlighting a need for assistance. Therefore, these associations underline the importance of addressing nutritional and weight-related factors in disability prevention strategies.

Recently, considerable attention has been directed towards the association between lifestyle behaviours, such as adherence to daily PA and nutrition recommendations and disability outcomes. Our study found a strong link between a lack of adherence to dietary recommendations and regular exercise, leading to a sharp decline in functional mobility. Regular participation in exercise among older adults has shown protection against falls and functional status decline [43–45]. For instance, engaging in a minimum of 150 min per week of moderately intense activity, such as brisk walking, could reduce the relative risk of losing functional independence by up to 30%, with an additional 30% reduction for more vigorous activities [45]. As such, previous studies have demonstrated the effectiveness of strength endurance training and exercise in improving muscular strength, balance, mobility, and physical function, highlighting the importance of exercise in promoting overall well-being in the older population [44, 46]. In addition, nutrition is also another modifiable risk factor that plays a crucial role in maintaining functional independence and preventing age-related chronic diseases. Several studies have demonstrated that adopting a healthy diet is linked to better health and a higher health-related quality of life in older adults [3, 5, 47, 48]. Therefore, these findings highlight that it is very important for policymakers to shift away from a healthcare system primarily focused on disease treatment and design person-centered, individualized disease prevention strategies.

Although the study findings are robust, some limitations should be noted. The usage of proxy interviews may have an impact on the accuracy of the results as ADL and IADL limitations questions are based on the experiences of the respondents themselves. Furthermore, telephone interviews and self-administered questionnaires in ATHIS 2014 were used for sensitive topics, which may have led to response bias. Due to the sampling survey design, there was a higher nonresponse rate among the older population, possibly leading to an overrepresentation of healthier participants. Nevertheless, the study also has several strengths. Data were collected from a representative sample of the Austrian population from two cross-sectional surveys with a 5-year gap. Additionally, in our analysis, we adjusted for various variables, including sociodemographic and health-related variables, which also helps to mitigate this potential bias and enhance the study’s robustness.

## Conclusion

Our study reveals a rising trend in the prevalence of disability among older adults in Austria. These findings offer valuable insights for healthcare providers and policymakers, guiding the development of future strategies for disability prevention and health promotion. In line with initiatives in other European countries, Austria must improve its preventive and long-term care services, with a particular focus on addressing the needs of women, individuals aged 80 years and above, individuals with primary education, those not in a relationship, those born outside EU/EFTA states, those with multiple chronic diseases, and individuals who do not comply with PA and daily nutritional recommendations.
